# Sequence and structure based models of HIV-1 protease and reverse transcriptase drug resistance

**DOI:** 10.1186/1471-2164-14-S4-S3

**Published:** 2013-10-01

**Authors:** Majid Masso, Iosif I Vaisman

**Affiliations:** 1 Laboratory for Structural Bioinformatics, School of Systems Biology, George Mason University, 10900 University Boulevard MS 5B3, Manassas, Virginia 20110, USA

## Abstract

**Background:**

Successful management of chronic human immunodeficiency virus type 1 (HIV-1) infection with a cocktail of antiretroviral medications can be negatively affected by the presence of drug resistant mutations in the viral targets. These targets include the HIV-1 protease (PR) and reverse transcriptase (RT) proteins, for which a number of inhibitors are available on the market and routinely prescribed. Protein mutational patterns are associated with varying degrees of resistance to their respective inhibitors, with extremes that can range from continued susceptibility to cross-resistance across all drugs.

**Results:**

Here we implement statistical learning algorithms to develop structure- and sequence-based models for systematically predicting the effects of mutations in the PR and RT proteins on resistance to each of eight and eleven inhibitors, respectively. Employing a four-body statistical potential, mutant proteins are represented as feature vectors whose components quantify relative environmental perturbations at amino acid residue positions in the respective target structures upon mutation. Two approaches are implemented in developing sequence-based models, based on use of either relative frequencies or counts of n-grams, to generate vectors for representing mutant proteins. To the best of our knowledge, this is the first reported study on structure- and sequence-based predictive models of HIV-1 PR and RT drug resistance developed by implementing a four-body statistical potential and n-grams, respectively, to generate mutant attribute vectors. Performance of the learning methods is evaluated on the basis of tenfold cross-validation, using previously assayed and publicly available *in vitro *data relating mutational patterns in the targets to quantified inhibitor susceptibility changes.

**Conclusion:**

Overall performance results are competitive with those of a previously published study utilizing a sequence-based strategy, while our structure- and sequence-based models provide orthogonal and complementary prediction methodologies, respectively. In a novel application, we describe a technique for identifying every possible pair of RT inhibitors as either potentially effective together as part of a cocktail, or a combination that is to be avoided.

## Background

With the advent of highly active antiretroviral therapy (HAART) for treating human immunodeficiency virus type 1 (HIV-1) infection, mortality rates from acquired immunodeficiency syndrome (AIDS) have significantly decreased in recent years [1]. HAART encompasses a variety of treatment strategies, each employing a distinct combination of at least three drugs designed to inhibit proteins essential to the viral replication cycle [2]. The HIV-1 protease (PR) and reverse transcriptase (RT) enzymes are critical targets of these drug cocktails, and the U.S. Food and Drug Administration (FDA) has approved a number of PR inhibitors (PIs) as well as nucleoside/nucleotide and nonnucleoside RT inhibitors (NRTIs and NNRTIs, respectively). Nevertheless, the evolution of drug resistant mutations in the PR and RT proteins poses a persistent risk to continued treatment success. The potential for any drug resistant mutation in either target to confer cross-resistance to other medications in the respective inhibitor class also raises a significant impediment to selecting optimal therapies. Consequently, a systematic understanding of how alternative mutational patterns in these target proteins affect susceptibility levels to their respective inhibitors is of vital importance in providing effective, personalized HAART regimens.

**Figure 1:**
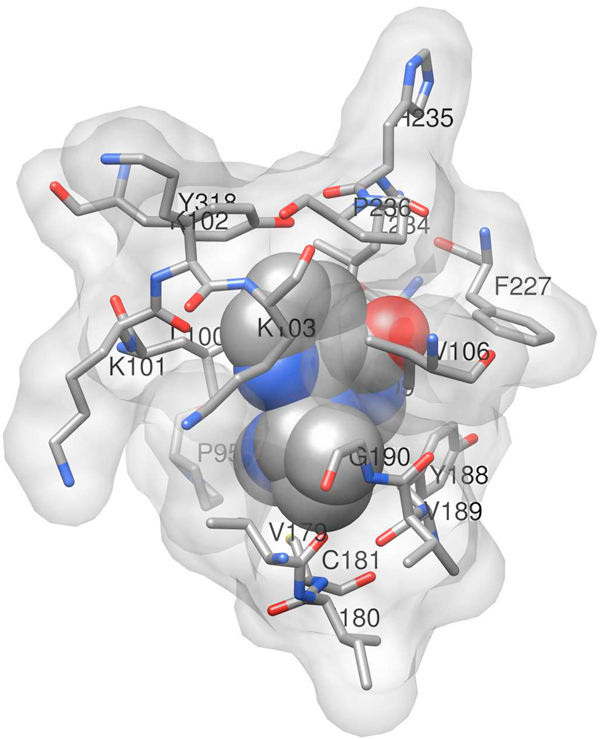
**Y181C mutant of HIV-1 RT in complex with the NNRTI nevirapine**. Shown are residues of the catalytic p66 subunit of RT that are within 5 angstroms of the inhibitor. Major mutations associated with nevirapine resistance occur at positions K103, V106, Y181, Y188, and G190; minor mutations occur at L100, K101, and several additional positions that are more distant from the inhibitor binding site. The diagram is based on atomic coordinates provided by Protein Data Bank (PDB) accession code 1jlb.

Of the three classes of HIV-1 drugs described above, PIs and NRTIs represent competitive inhibitors designed to bind relatively conserved active sites of the HIV-1 PR and RT enzymes. On the other hand, NNRTIs are non-competitive inhibitors that bind a less conserved hydrophobic pocket of RT near the active site (Figure 1) [3], resulting in conformational changes to the enzyme that prevent its polymerization activity. Amino acid substitutions in the PR and RT proteins associated with drug resistance fall into two general categories: major and minor [4]. Major mutations are single residue replacements that alone are capable of significantly decreasing the susceptibility to one or more drugs in a particular class, they generally occur either at positions forming the inhibitor binding site or at nearby positions affecting its geometry, and they frequently appear in clinical samples sequenced from patients experiencing virologic failure. Substrate binding and catalytic activity of the PR and RT enzymes are negatively impacted by major mutations associated with inhibitors that bind the protein active sites. Subsequently, minor mutations may appear either to increase marginally the level of drug resistance (accessory), or to create structural rearrangements that help restore enzyme activity and improve viral fitness (compensatory) [5]. Minor mutations may appear either near the substrate or inhibitor binding sites, or they may exert their effects allosterically from structurally distant positions. A number of natural polymorphisms in untreated patients that may slightly increase drug resistance are also referred to as minor mutations.

Genotype tests are available for rapidly and inexpensively discerning whether mutations already known to be associated with inhibitor resistance are present in HIV-1 PR and RT sequences. Relatively more time consuming and costly phenotype testing, on the other hand, quantitatively measures the change in susceptibility of a mutant PR or RT target protein to an inhibitor relative to that of a drug-sensitive control. Hence, a number of algorithmic approaches have been developed over the past decade for the prediction of phenotype from genotype [6-10], including models trained using statistical machine learning techniques [11-13]. Studies have alternatively focused on structure-based prediction of resistance patterns, using approaches as varied as molecular modeling [14,15], fitness evolution [16], molecular dynamics simulations [17], and statistical learning [18-21]. Additionally, recent efforts have evaluated the inclusion of clinical data based on known patient outcomes as supplementary attributes [22,23].

Employing a sequence-based approach, Rhee *et al. *[24] previously applied five statistical learning methods to sets of HIV-1 PR and RT mutants available from the Stanford University HIV Drug Resistance Database [25] in order to systematically develop predictive models of resistance to each of 16 inhibitors. The goal of the present study is to implement distinct structure and sequence based approaches, previously not considered in this arena, and to apply four learning methods (random forest, RF; support vector machine, SVM; reduced-error pruned tree regression, REPTree; and support vector regression, SVR), in order to develop and compare a comprehensive set of predictive models of PR and RT resistance to 19 antiretroviral drugs (8 PIs, 8 NRTIs, and 3 NNRTIs). For model training and testing we rely on updated versions of the same datasets from the Stanford Database as those that were employed in the key study of Rhee *et al. *[24].

As detailed in the Methods, our structure-based technique involves threading of mutant amino acid sequences onto native PR and RT structures obtained from the Protein Data Bank (PDB) [26], as well as application of a computational mutagenesis methodology incorporating a four-body, knowledge-based, statistical potential energy function [27]. For each PR or RT mutational pattern, this approach allows us to quantify both an overall change in protein sequence-structure compatibility relative to wild-type (*residual score*) as well as ensuing environmental perturbations (*EC scores*) at all constituent amino acid positions in the target protein structure, the latter of which define attributes for a feature vector representation of the mutant. Our performance measures are similar to those reported by the Stanford group in Rhee *et al. *[24], despite the relatively smaller sizes of our training sets, with both techniques representing orthogonal prediction strategies. Lastly, we summarize results of complementary sequence-based models that utilize previously unexplored attributes for mutant PR and RT feature vectors obtained through two applications of n-grams (subsequences of size *n*), which we refer to as relative frequency and counts methods [28].

## Results and discussion

### Inhibitor datasets

Brand names and abbreviations for the inhibitors under consideration are provided in Table 1 (adapted from [28]), along with the distribution of sensitive (S), intermediate (I), and resistant (R) susceptibilities for each corresponding dataset of mutants. Sixteen of our 19 inhibitor datasets overlap with those of the sequence-based study by the Stanford group (Table 2 in [24]; inhibitors TPV, ddC, and FTC were not included in that study), though the total number of mutants in each of our datasets is substantially lower since we exclude all isolates displaying electrophoretic mixtures of amino acids at any HIV-1 PR or RT sequence position, whereas Rhee *et al*. only excluded isolates with mixtures occurring at nonpolymorphic drug resistance positions in those proteins. Despite absolute size differences between comparable pairs of inhibitor datasets in our work and in the study by the Stanford group, the distribution of mutants across each of the three drug susceptibility categories over these 16 pairs of datasets are highly correlated (concordance correlation coefficients [29]: S, *r*_c _= 0.86; I, *r*_c _= 0.90; and R, *r*_c _= 0.96), suggesting similarly stratified datasets in both studies.

**Table 1:** Distribution of mutant HIV-1 isolates by inhibitor susceptibility

	Isolate Phenotypes (%) ^a^			
		
Drug	S	I	R	Total
	Protease Inhibitors			
Amprenavir (APV)	63	26	11	495
Atazanavir (ATV)	49	29	22	200
Indinavir (IDV)	53	26	21	502
Lopinavir (LPV)	46	22	32	320
Nelfinavir (NFV)	39	28	33	526
Ritonavir (RTV)	50	20	30	473
Saquinavir (SQV)	61	18	21	509
Tipranavir (TPV)	78	11	11	47
	Nucleoside/Nucleotide RT Inhibitors			
Lamivudine (3TC)	29	18	53	244
Abacavir (ABC)	28	45	27	237
Zidovudine (AZT)	50	23	27	240
Stavudine (d4T)	53	36	11	242
Zalcitabine (ddC)	39	52	9	161
Didanosine (ddI)	51	43	6	243
Emtricitabine (FTC)	31	13	56	52
Tenofovir (TDF)	65	25	10	167
	Nonnucleoside RT Inhibitors			
Delavirdine (DLV)	53	20	27	304
Efavirenz (EFV)	53	22	25	296
Nevirapine (NVP)	43	11	46	307

^a ^S, sensitive; I, intermediate; R, resistant. Category thresholds are discussed in Methods.

### Structure-function relationships

**Figure 2:**
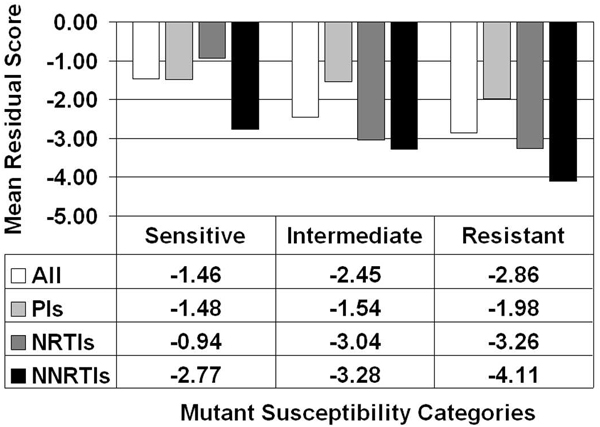
**Elucidation of structure-function relationships in HIV-1 PR and RT**. Residual scores of mutant proteins in the inhibitor datasets quantify sequence-structure compatibility changes, whereas corresponding mutant susceptibilities to inhibitors identify functional consequences. Results averaged over datasets comprising each inhibitor class (PIs/NRTIs/NNRTIs) separately, as well as collectively over all classes.

Implementation of our computational mutagenesis technique as detailed in the Methods allowed for the calculation of residual scores for all the HIV-1 PR and RT mutants, which we used to obtain a mean residual score by susceptibility category (S/I/R) within each of the 19 inhibitor datasets. For a given susceptibility category, the overall average was determined for the associated mean residual scores over all 19 inhibitor datasets (Figure 2, All), as well as separate averages only over those datasets that belong to each inhibitor class (Figure 2, PIs/NRTIs/NNRTIs). A clear trend based on these data is evident in Figure 2, whereby mean residual scores decrease with increasing resistance. Given the quantitative definition of a mutant residual score and its interpretation, the results suggest that PR and RT mutants having increasingly detrimental effects on protein sequence-structure compatibility are also those that are more likely to be associated with a greater degree of drug resistance.

### Structure-based mutant attributes and statistical learning techniques

Following a procedure similar to that described in Rhee *et al. *[24], HIV-1 PR or RT mutants comprising the inhibitor datasets are represented as training sets of feature vectors for the learning algorithms based on three distinct sets of attributes as components (Table 2, adapted and modified from [24]). The first approach makes use of the entire residual profile (EC scores at all 99 PR or 543 RT positions, All) for each mutant. Next, we consider mutant feature vectors whose components consist of the EC scores only at PR or RT positions for which residue substitutions occur that, according to an expert panel (International Antiviral Society - USA, IAS), are associated with drug resistance [4,30]. Finally, we evaluate the utility of mutant feature vectors whose components consist of the EC scores at PR or RT positions for which residue substitutions occur that are significantly more common in treated versus untreated individuals (nonpolymorphic treatment-selected mutations, TSM) [31].

**Table 2:** Sets of HIV-1 PR and RT residue positions used to construct feature vectors

Set^a^	Description	Positions
All	EC scores at all residue positions in structures for HIV-1 PR (PDB ID: 3phv) and RT (PDB ID: 1rtj, chain A)	PIs: 1 - 99 NRTIs and NNRTIs: 1 - 543
IAS	EC scores only at positions for which residue substitutions occur that are associated with drug resistance.	PIs (common to all: 10, 82, 84, 90) APV: 32, 46, 47, 50, 54, 73, 76 ATV: 16, 20, 24, 32-34, 36, 46, 48, 50, 53, 54, 60, 62, 64, 71, 73, 85, 88, 93 IDV: 20, 24, 32, 36, 46, 54, 71, 73, 76, 77 LPV: 20, 24, 32, 33, 46, 47, 50, 53, 54, 63, 71, 73, 76 NFV: 30, 36, 46, 71, 77, 88 RTV: 20, 32, 33, 36, 46, 54, 71, 77 SQV: 24, 48, 54, 62, 71, 73, 77 TPV: 13, 20, 33, 35, 36, 43, 46, 47, 54, 58, 69, 74, 83 NRTIs (common to all: 41, 67, 70, 210, 215, 219) 3TC: 62, 65, 75, 77, 116, 151, 184 ABC: 62, 65, 74, 75, 77, 115, 116, 151, 184 AZT: 62, 75, 77, 116, 151 d4T: 62, 65, 75, 77, 116, 151 ddC: 62, 65, 69, 74, 75, 77, 116, 151, 184 ddI: 62, 65, 74, 75, 77, 116, 151 FTC: 62, 65, 75, 77, 116, 151, 184 TDF: 65 NNRTIs (common to all: 100, 103, 106, 181, 188, 190) DLV: 230, 236 EFV: 101, 108, 225 NVP: 101, 108
TSM	EC scores only at positions for which residue substitutions occur that are significantly more common in treated versus untreated individuals.	PIs: 10, 11, 20, 23, 24, 30, 32-35, 43, 46-48, 50, 53-55, 58, 66, 67, 71, 73, 74, 76, 79, 82, 84, 85, 88-90, 92, 95 NRTIs: 41, 43, 44, 62, 65, 67, 69, 70, 74, 75, 77, 98, 115, 116, 151, 184, 203, 208, 210, 215, 218, 219, 223, 228 NNRTIs: 100, 101, 103, 106, 108, 138, 181, 188, 190, 221, 225, 227, 230, 236, 238

^a ^IAS, International Antiviral Society; TSM, nonpolymorphic treatment selected mutations.

Implementations of two classification [random forest (RF) and support vector machine (SVM)] and two regression [reduced-error pruned tree (REPTree) regression and support vector regression (SVR)] statistical learning methods are used in conjunction with each of the three representations of our 19 inhibitor datasets of HIV-1 PR and RT mutants as described in the previous paragraph. For a given inhibitor dataset of mutants, the RF and SVM methods predict the susceptibility of each mutant to that inhibitor as sensitive (S), intermediate (I), or resistant (R). On the other hand, the regression methods predict for each PR or RT mutant a numerical value for the change in susceptibility to the inhibitor relative to that of a drug-sensitive, wild-type protein; subsequently, these predicted values are used for classifying the mutants based on category thresholds.

### Structure-based classification and regression summaries

Mean prediction accuracies (4 learning methods × 3 attribute datasets each) are equivalent for the PIs (78.0%), NRTIs (77.2%), and NNRTIs (78.3%), with no single inhibitor class displaying a selective advantage over another (PIs-NRTIs, *p *= 0.17; PIs-NNRTIs, *p *= 0.40; NRTIs-NNRTIs, *p *= 0.15) (Table 3). Comparisons with results reported in the sequence-based study by the Stanford group (Table 3 in [24]: PIs, 78.2%; NRTIs, 75.9%; and NNRTIs, 83.0%) reveals statistically significant differences for the latter two classes of inhibitors (PIs, *p *= 0.45; NRTIs and NNRTIs, *p *< 0.05), reflecting our prediction superiority with the NRTIs and that of the Stanford group with the NNRTIs. Inhibitors displaying the highest and lowest mean accuracy, respectively by class, in Table 3 of our study are ritonavir (82.7%) and atazanavir (69.3%) for the PIs; emtricitabine (89.4%) and tenofovir (69.6%) for the NRTIs; and nevirapine (81.5%) and delavirdine (74.8%) for the NNRTIs. These results mirror those reported by the Stanford group, with the exception that in their work, the NRTI lamivudine took the place of the newer and structurally similar emtricitabine, since data for the latter drug was not available at the time; in our study, lamivudine displays the second highest mean accuracy (84.8%) after emtricitabine among the NRTIs.

**Table 3:** Predictive accuracy of REPTree, SVR, RF, and SVM using TSM, All, and IAS sets to construct mutant feature vectors

	REPTree			SVR			RF			SVM			
					
Drug	TSM	All	IAS	TSM	All	IAS	TSM	All	IAS	TSM	All	IAS	DrugMean
Protease Inhibitors													
APV	0.79	0.80	0.78	0.77	0.78	0.76	0.78	0.79	0.78	0.77	0.77	0.75	0.78
ATV	0.69	0.69	0.71	0.69	0.67	0.67	0.74	0.74	0.74	0.67	0.65	0.65	0.69
IDV	0.77	0.78	0.76	0.78	0.78	0.76	0.77	0.78	0.78	0.76	0.76	0.76	0.77
LPV	0.78	0.78	0.79	0.72	0.72	0.72	0.80	0.82	0.80	0.72	0.72	0.72	0.76
NFV	0.80	0.81	0.78	0.78	0.78	0.78	0.80	0.82	0.79	0.78	0.78	0.78	0.79
RTV	0.86	0.87	0.83	0.80	0.82	0.76	0.86	0.86	0.86	0.80	0.79	0.81	0.83
SQV	0.80	0.80	0.79	0.82	0.82	0.80	0.81	0.82	0.82	0.81	0.82	0.81	0.81
TPV	0.83	0.81	0.79	0.79	0.87	0.85	0.79	0.79	0.79	0.81	0.81	0.91	0.82
AVG	0.79	0.79	0.78	0.77	0.78	0.76	0.79	0.80	0.80	0.77	0.76	0.77	0.78
Nucleoside/Nucleotide RT Inhibitors													
3TC	0.89	0.89	0.89	0.68	0.86	0.69	0.90	0.89	0.89	0.86	0.87	0.86	0.85
ABC	0.71	0.68	0.71	0.70	0.71	0.68	0.71	0.73	0.72	0.68	0.65	0.67	0.70
AZT	0.69	0.73	0.73	0.78	0.76	0.73	0.74	0.75	0.74	0.72	0.78	0.72	0.74
d4T	0.72	0.75	0.73	0.77	0.77	0.76	0.79	0.76	0.74	0.79	0.76	0.78	0.76
ddC	0.77	0.79	0.78	0.79	0.76	0.78	0.80	0.77	0.83	0.78	0.78	0.79	0.79
ddI	0.78	0.76	0.75	0.74	0.77	0.73	0.75	0.76	0.74	0.77	0.75	0.76	0.76
FTC	0.92	0.94	0.92	0.81	0.81	0.94	0.94	0.94	1.00	0.83	0.83	0.85	0.89
TDF	0.73	0.72	0.69	0.69	0.69	0.65	0.73	0.76	0.68	0.67	0.67	0.67	0.70
AVG	0.78	0.78	0.78	0.75	0.77	0.75	0.80	0.80	0.79	0.76	0.76	0.76	0.77
Nonnucleoside RT Inhibitors													
DLV	0.74	0.71	0.75	0.76	0.70	0.76	0.76	0.75	0.74	0.78	0.74	0.78	0.75
EFV	0.79	0.79	0.79	0.79	0.75	0.74	0.85	0.81	0.84	0.78	0.74	0.76	0.79
NVP	0.83	0.83	0.83	0.82	0.69	0.79	0.85	0.84	0.86	0.83	0.76	0.85	0.82
AVG	0.79	0.78	0.79	0.79	0.71	0.76	0.82	0.80	0.81	0.80	0.75	0.80	0.78

Averaging over 19 inhibitors and three attribute datasets, the mean accuracies of the learning methods are 79.8% for RF, 78.3% for REPTree, 76.7% for SVM, and 76.0% for SVR. This is due to the fact that with respect to each of the three attribute datasets individually, RF significantly outperforms each of the other three learning methods; for example, based only on datasets using EC scores at all positions (Table 3, "All" columns), the mean accuracy of RF (79.9%) is significantly higher than those of REPTree (78.6%, *p *< 0.01), SVR (76.4%, *p *< 0.01), or SVM (75.9%, *p *< 0.001). Classification methods (79.6%) display higher accuracy for the NNRTIs than regression methods (77.0%, *p *< 0.05); however, no such differences are observed between the methods for the PIs or NRTIs. Finally, averaging over all 19 inhibitors and four learning methods, no statistically significant differences are seen between the mean accuracies of the TSM (77.8%), All (77.7%), and IAS (77.6%) attribute datasets (TSM-All, *p *= 0.37; TSM-IAS, *p *= 0.24; All-IAS, *p *= 0.41).

In addition to the overall prediction accuracy, we calculated the balanced error rate (BER) and the area (AUC) under the receiver operating characteristic (ROC) curve for the RF and SVM classification methods (Additional file 1). Averaged over all 19 inhibitors and three attribute datasets, the mean BER and AUC values for RF (0.29 and 0.91, respectively) are superior to those for SVM (0.31 and 0.86); however, only AUC differences are statistically significant (*p *< 0.001), a result which also holds for each attribute dataset separately. Next, averaged over all attribute datasets and both learning methods, the mean BER and AUC values for the PIs (0.31 and 0.91, respectively), NRTIs (0.28 and 0.87), and NNRTIs (0.32 and 0.88) reflect no statistically significant BER differences between any pair of inhibitor classes. On the other hand, statistically significant AUC differences exist between the PIs and each of the other inhibitor classes (PIs-NRTIs, *p *< 0.01; PIs-NNRTIs, *p *< 0. 01; NRTIs-NNRTIs, *p *= 0.33). The TSM datasets yield mean BER and AUC values (0.29 and 0.90, respectively) that are superior to those of the All (0.31 and 0.89, respectively) and IAS (0.29 and 0.88) datasets when averaged over all 19 inhibitors and both learning methods, though the differences are not significant. Lastly, out-of-bag (OOB) errors associated with all RF classifications are tabulated (Additional file 2) and display no statistically significant differences when averaged over either the inhibitor classes or the attribute datasets.

Considering all 19 inhibitors as well as both the REPTree and SVR regression methods, TSM datasets display the highest overall correlation coefficients (*r*^2^) between actual and predicted relative susceptibility levels of the constituent HIV-1 PR and RT mutants (Additional file 3), a result similarly reported by the Stanford group (Table 6 in [24], available as online supporting information). However, overall differences between our three attribute datasets are minimal (averaged *r*^2 ^values: TSM, 0.71; All, 0.70; and IAS, 0.68). For the TSM datasets, our averaged *r*^2 ^values obtained from both regression methods are 0.69 over the PIs, 0.73 over the NRTIs, and 0.70 over the NNRTIs. Over the 19 inhibitors and both regression methods, our averaged mean-squared error (mse) values are 0.22 for TSM, 0.24 for All, and 0.23 for IAS datasets (Additional file 3), results which coincide with those of the Stanford group for the TSM datasets (Table 7 in [24], available as online supporting information); however with respect to the IAS datasets, our averaged mse values are substantially lower than those of the Stanford group (0.23 versus 0.32). For our TSM datasets, the averaged mse values over both regression methods vary considerably among the inhibitor classes (0.27 for the PIs, 0.10 for the NRTIs, and 0.43 for the NNRTIs).

### Influence of dataset size on performance of structure-based models

Using inhibitor datasets that contain significantly fewer mutants, our prediction accuracies are generally comparable to those of the sequence-based method in Rhee *et al. *[24], suggesting that our structure-based attributes encode a greater degree of information content. Averaged over all 16 inhibitors common to both studies, our datasets combined consist of fewer than half (49.9%) the number of mutants used by the Stanford group, with a maximum of 64.4% for the PI amprenavir and a minimum of 37.7% for the NRTI abacavir.

**Figure 3:**
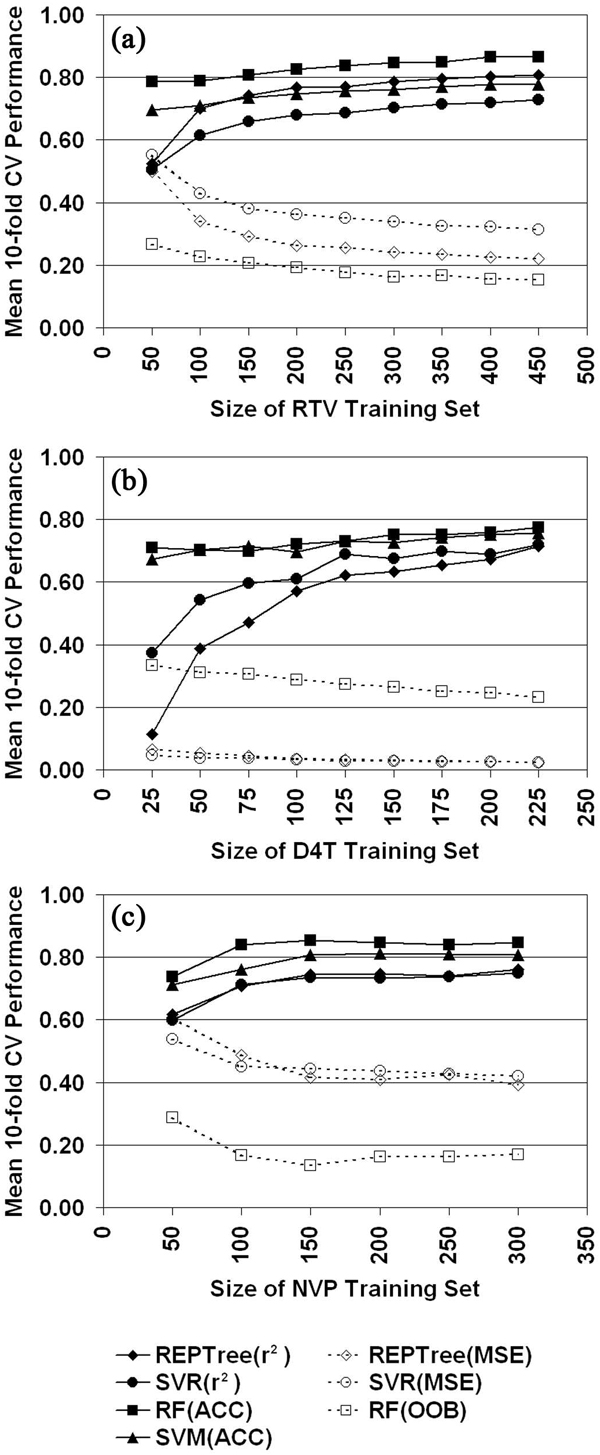
**Relating dataset size to model performance**. Learning curves plotted for (a) ritonavir (RTV), (b) stavudine (d4T), and (c) nevirapine (NVP) using their respective TSM attribute mutant datasets (ACC, overall accuracy).

To better understand the influence of dataset size on model performance, we generated learning curves (Figure 3) by using the TSM attribute datasets and selecting one representative drug from each inhibitor class: ritonavir for the PIs, stavudine for the NRTIs, and nevirapine for the NNRTIs. The plots evaluate all four learning methods and were generated by initially applying tenfold cross-validation (10-fold CV) testing to each of 10 stratified random samples of 50 mutants (25 mutants for stavudine) selected with replacement from their respective inhibitor datasets. After calculating mean 10-fold CV performance measures, subsequent iterations were carried out which involved incrementing by 50 mutants (25 for stavudine) the sizes of the sampled subsets. The learning curves of Figure 3 suggest that sample sizes significantly affect the performance of the statistical learning methods, a result similar to one reported in Rhee *et al. *[24].

### Structure-based prediction of paired HIV-1 RT drug effects

The reported classification and regression summary data support the finding that TSM attribute datasets consistently outperform the All and IAS datasets in predicting mutant HIV-1 PR or RT susceptibility to inhibitors, albeit marginally in some cases. Similar trends were reported by the Stanford group, where they reasonably postulated that prediction success using TSMs emerged from selective pressure on HIV-1 to escape drug inhibition [24]. Successful HAART regimens either target HIV-1 on multiple fronts (e.g., PR and RT enzymes), or they consist of medications that display non-overlapping mutational patterns of cross-resistance (e.g., NRTIs and NNRTIs are associated with TSMs at disparate subsets of RT residue positions; see Table 2). Specifically, many cocktails include a pair of NRTIs that display incongruent patterns of selective mutational pressure across the TSM positions for the class, and these are typically combined with an NNRTI (or a PI) [2]. In addition to the potential for cross-resistance, serious factors that may preclude the pairing of certain drugs include antagonism, toxicity, and reduced efficacy.

An application of our structure-based approach facilitates the identification of potentially effective drug combinations targeting HIV-1 RT. First, we represent the RT mutants comprising each of the NRTI and NNRTI inhibitor datasets as feature vectors consisting of EC score attributes at all 39 combined TSM residue positions associated with both inhibitor classes (Table 2: 24 and 15 TSM positions associated with NRTIs and NNRTIs, respectively). Next, we select one inhibitor dataset of RT mutants to train a REPTree regression model, and testing is performed on another dataset. All possible pairs of datasets are used as training/testing combinations, and we report the correlation coefficients (-1 ≤ *r *≤ 1) between the actual and predicted relative susceptibility levels of the mutants in each test set; in cases where the same inhibitor is used for training and testing, the results reflect the resubstitution error for that dataset (Table 4). Positive values of *r *→ 1 suggest a greater likelihood of 1) similar mutant susceptibility profiles and consequent cross-resistance between the inhibitors representing the training/test set pair; or 2) increased antagonism and toxicity, or reduced efficacy with respect to concomitant administration of both drugs. On the other hand, effective drug combinations are more likely to be associated with training/test set pairs that yield insignificant (relatively closer to zero) or negative correlations.

**Table 4:** REPTree regression correlation coefficients (*r*) using TSM positions for both NRTIs and NNRTIs to construct structure-based mutant feature vectors

	NRTIs								NNRTIs		
		
Train/Test	3TC	ABC	AZT	d4T	ddC	ddI	FTC	TDF	DLV	EFV	NVP
NRTIs											
3TC	0.99	0.71	-0.05	0.04	0.45	0.39	1.00	-0.29	-0.11	-0.16	-0.23
ABC	0.83	0.92	0.28	0.44	0.64	0.67	0.91	0.02	-0.13	-0.08	-0.17
AZT	0.07	0.39	0.90	0.75	0.17	0.28	0.34	0.63	-0.03	0.02	-0.01
d4T	0.17	0.55	0.76	0.93	0.56	0.64	0.36	0.56	-0.11	-0.04	-0.08
ddC	0.57	0.67	0.15	0.48	0.90	0.85	0.93	-0.03	-0.14	-0.14	-0.20
ddI	0.41	0.69	0.28	0.63	0.85	0.92	0.70	0.13	-0.12	-0.06	-0.12
FTC	0.94	0.67	-0.02	0.02	0.42	0.35	0.99	-0.30	-0.15	-0.19	-0.26
TDF	-0.46	-0.03	0.68	0.57	-0.08	0.04	-0.41	0.86	0.04	0.09	0.10
NNRTIs											
DLV	-0.15	-0.12	0.08	0.01	-0.08	-0.09	-0.33	-0.01	0.89	0.60	0.68
EFV	-0.09	-0.01	0.12	0.04	-0.09	-0.04	-0.13	0.04	0.64	0.93	0.79
NVP	-0.13	-0.05	0.16	0.08	-0.13	-0.09	-0.21	0.03	0.63	0.72	0.93

Given the interpretations outlined above, the data in Table 4 reflect both the historical spectrum of clinically prescribed drug cocktails as well as the currently recommended treatment guidelines [2]. Our complementary sequence-based (n-grams) models yield a similar table of correlations published in a recent report [28], where a subsequent discussion detailing specifically how those data mirror the treatment guidelines also applies here to the structure-based results of Table 4.

## Sequence-based classification and regression summaries

As fully described in our companion study [28], the HIV-1 PR and RT mutants comprising the 19 inhibitor-specific datasets are represented as feature vectors of sequence-based input attributes through two types of n-grams applications, referred to as the relative frequency and the counts methods, and these datasets are used in conjunction with two statistical learning algorithms (REPTree regression and RF classification). Our sequence-based results (Table 5 adapted from [28]) are in line with those of both our structure-based models as well as the sequence-based models of the Stanford group with respect to the PI and NNRTI classes, and similar to what was observed with our structure-based models, this n-grams approach ranks lamivudine second to emtricitabine in mean accuracy among the NRTIs. A minor discrepancy is observed, whereby our sequence-based models rank tenofovir second lowest in mean accuracy compared to abacavir among the NRTIs, while both our structure-based models and the sequence-based models of the Stanford group have these rankings inverted. Additional performance data for our sequence-based models and a detailed analysis of all results are provided in the aforementioned report.

**Table 5:** Predictive accuracy of REPTree and RF using relative frequency and counts methods to represent dataset sequences

	Relative Frequency		Counts		
			
Drug	REPTree	RF	REPTree	RF	Drug Mean
Protease Inhibitors					
APV	0.81	0.80	0.80	0.80	0.80
ATV	0.74	0.75	0.76	0.76	0.75
IDV	0.78	0.80	0.75	0.80	0.78
LPV	0.80	0.82	0.80	0.81	0.81
NFV	0.80	0.80	0.79	0.82	0.80
RTV	0.87	0.86	0.87	0.84	0.86
SQV	0.80	0.79	0.80	0.80	0.80
TPV	0.75	0.79	0.75	0.81	0.78
AVG	0.79	0.80	0.79	0.81	0.80
Nucleoside/Nucleotide RT Inhibitors					
3TC	0.89	0.87	0.87	0.90	0.88
ABC	0.68	0.68	0.66	0.67	0.67
AZT	0.75	0.75	0.73	0.70	0.73
d4T	0.74	0.79	0.76	0.78	0.77
ddC	0.80	0.75	0.80	0.76	0.78
ddI	0.69	0.73	0.69	0.71	0.71
FTC	0.96	0.83	0.94	0.89	0.91
TDF	0.75	0.75	0.68	0.74	0.73
AVG	0.78	0.77	0.77	0.77	0.77
Nonnucleoside RT Inhibitors					
DLV	0.76	0.70	0.76	0.71	0.73
EFV	0.78	0.74	0.76	0.73	0.75
NVP	0.84	0.79	0.82	0.77	0.81
AVG	0.79	0.74	0.78	0.74	0.76

## Sequence contribution to n-grams prediction

As shown in [28], we examine attributes selected at REPTree nodes for our sequence-based models in order to identify the most information-rich HIV-1 PR and RT residue positions used by those regression trees, thereby leading to their predictive capability. For each inhibitor-specific model, Table 6 (adapted from [28]) summarizes the attributes selected for the root node (most informative) as well as for nodes at the next two levels, where an attribute *i *corresponds to both sequence positions *i *and *i *+ 1 in either PR or RT based on the relative frequency n-grams approach (*n *= 2). Note that all root node attributes correspond to sequence positions that appear in both the IAS and TSM drug resistance mutation sets, while the majority (> 75%) of attributes at the next two levels of nodes correspond to positions that either also overlap both sets or appear exclusively in the TSM subsets.

**Table 6:** Feature vector attribute selections by REPTree regression models using relative frequency method

Drugs	Root Node ^a^	Level 1 Nodes ^a^	Level 2 Nodes ^a^
**PIs**			
APV:	10	84, **87**	32, **34**, 53
ATV:	54	73	32, 50
IDV:	54	45, 53	72, 83, 90
LPV:	54	45	77, 84
NFV:	10	**54**, 87	29, **75**, 83, 90
RTV:	54	9, 84	19, 82, 84
SQV:	70	10, 83	47, 54, 90
TPV:	90	**52**, 56	40, 73
**NRTIs**			
3TC:	183	64	66
ABC:	183	115, 214	64, 101, 114, 118
AZT:	67	166, 210	76, 214
d4T:	209	76, 177	66, 67
ddC:	115	134, 183	65, 117
ddI:	150	**43**, 61	39, **183**
FTC:	183	123, 214	40
TDF:	214	34, 65	**68**, **227**, 285
**NNRTIs**			
DLV:	102	165, 180	69, 100, 190, 209
EFV:	102	189	99, 188
NVP:	189	103, 172	173, 180

^a ^Regular font, both IAS and TSM sets of positions; bold, TSM set only; underlined, neither set.

## Conclusions

**Figure 4:**
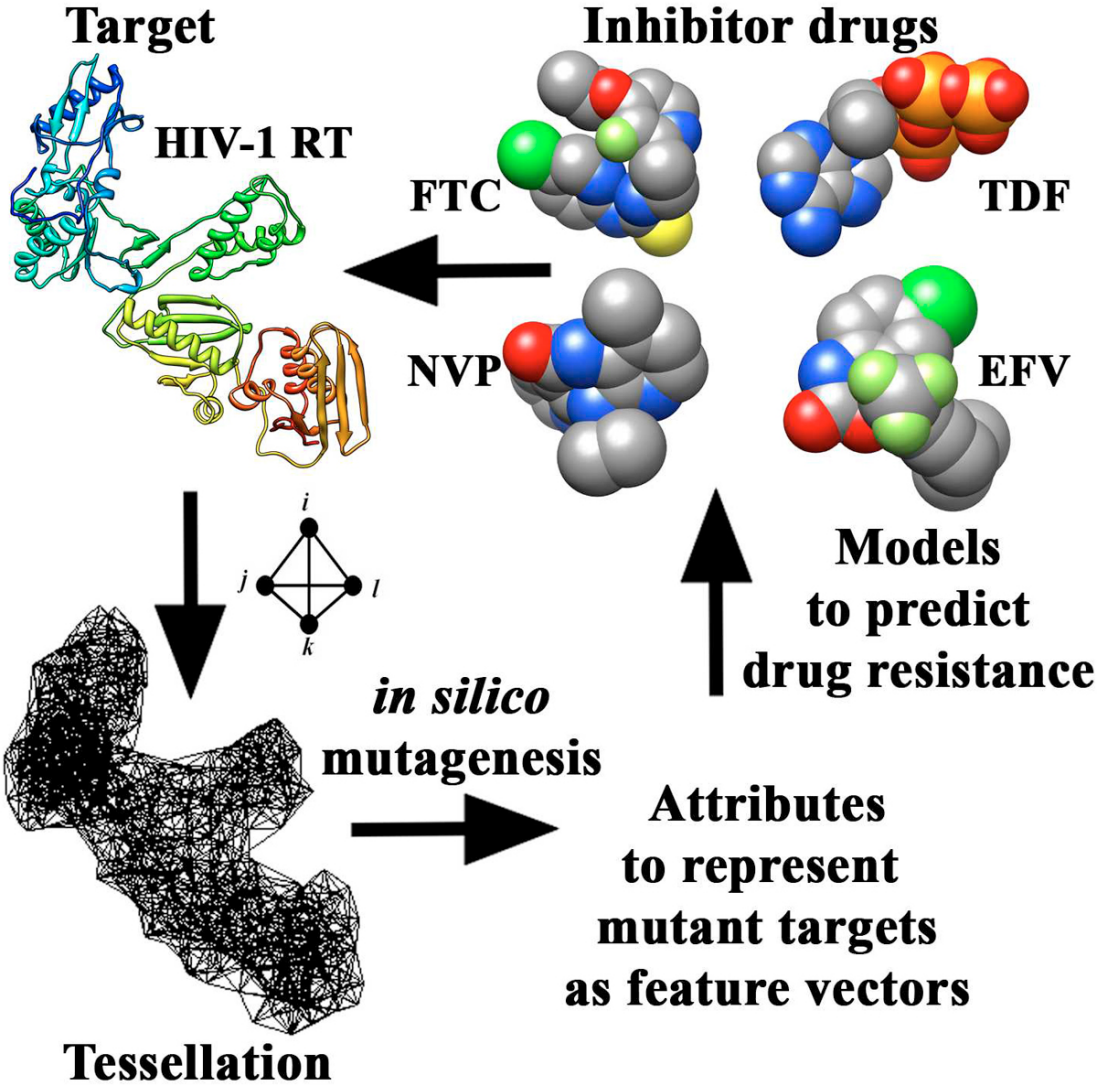
**Graphical summary of the structure-based study methodology**. A structure-based approach makes use of a computational mutagenesis methodology to generate attributes for feature vectors representing HIV-1 RT mutants. Mutants with known phenotypes (levels of susceptibility to various inhibitor drugs) are used to train predictive classification and regression models of drug resistance.

We developed accurate and efficient statistical learning models, based on innovative approaches using structure and sequence, for systematically relating residue replacements in HIV-1 PR and RT target enzymes (genotypes) to quantified changes in susceptibility to each of 19 antiretroviral drugs (phenotypes). The models were trained using datasets of previously assayed mutants with known phenotypes that were made available from the Stanford University HIV Drug Resistance Database [25]. For the structure-based models, mutant PR or RT proteins were represented as feature vectors whose input attributes, obtained via an *in silico *mutagenesis technique relying on a four-body statistical potential energy function, quantified environmental perturbations at positions in their respective targets upon mutation (Figure 4). Similarly, we developed sequence-based models by generating mutant attributes through two applications of n-grams, a technique previously used by other groups in a variety of studies on proteins [32-36] though not in this particular realm. Our models display performance measures that are generally competitive with those described by Rhee *et al. *[24] in their seminal systematic study that utilizes a sequence-based approach. The structure-based (sequence-based) methods reported here represent prediction strategies that are orthogonal (complementary) to those of Rhee *et al*., and both studies employ non-overlapping, information-rich attributes. In a novel application, our models were used to classify all pairs of RT inhibitors as either potentially effective as part of an antiretroviral cocktail, or a combination that should not be concomitantly administered.

## Methods

### Datasets

All HIV-1 PR and RT mutational patterns and corresponding drug susceptibilities were obtained from isolates tabulated in the Stanford University HIV Drug Resistance Database [25]. Here we provide a brief summary of directly relevant information, with additional details reported by the Stanford group in Rhee *et al. *[24]. We excluded all isolates with electrophoretic evidence of multiple amino acids at one or more of the sequence positions 1 - 99 of PR or 1 - 543 of RT, causing our pool of available isolates to be substantially smaller than that of the Stanford group. Mutational patterns in PR or RT sequences, defined as amino acid substitutions at one or more positions and exclusive of any indels (insertions or deletions), were identified relative to comparisons with the HIV-1 subtype B consensus wild-type sequence. These PR and RT sequences correspond to mutant proteins for which susceptibility to one or more of their respective inhibitors were obtained using the PhenoSense assay (Monogram Biosciences, South San Francisco, CA) [37,38]. For each PR or RT mutant isolate, the assay reports susceptibility to an inhibitor as a fold change, defined as the ratio of 50% inhibitory concentration (IC_50_) for the mutant relative to that for a drug-sensitive, wild-type control.

Separate datasets were produced for the 19 HIV-1 PR and RT inhibitors under consideration, each consisting of all respective PR or RT mutant proteins for which fold change susceptibility values to the particular inhibitor are available. The mutants in each dataset were classified into three categories (sensitive, intermediate, resistant) based on previously reported fold change threshold values, and all fold change values were log-transformed and standardized prior to analysis [24,28,39].

### Computational mutagenesis methodology

**Figure 5:**
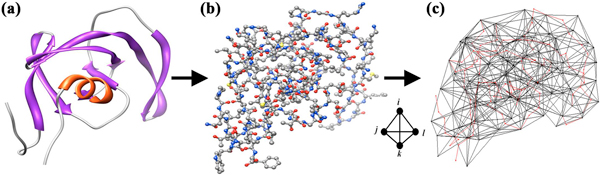
**Delaunay tessellation of HIV-1 PR**. (a) Ribbon and (b) ball and stick diagrams depicting the 99-residue single chain of HIV-1 PR are based on atomic coordinates provided by PDB accession code 3phv. (c) Delaunay tessellation of HIV-1 PR is superimposed over a Cα trace of the protein (drawn in red). The PR tessellation was generated using the center of mass coordinates of the amino acid side chains (Cα for glycine), which are represented by the tetrahedral vertices.

Structural coordinates for native HIV-1 PR and RT proteins were obtained from the Protein Data Bank (PDB accession codes: 3phv for PR; and 1rtj, chain A for RT) [26], which were used for generating residue-based coarse-grained representations of the proteins as collections of points in three-dimensional (3D) space, corresponding to centers of mass of the constituent amino acid residue side chains (Cα coordinates used for glycine). A convex hull of space-filling, non-overlapping, irregular tetrahedra was generated for each protein with the Qhull [40] implementation of the Delaunay tessellation algorithm, a classical computational geometry tiling technique [41], whereby the points serve as tetrahedral vertices (Figure 5). Hundreds of tetrahedra are typically generated by the tessellation of an average sized protein, each objectively identifying at its four vertices a quadruplet of structurally nearest neighbor residues. Assuming an *N *= 20 letter amino acid alphabet, the *r *= 4 vertices of each tetrahedron may correspond to any one of

$$\left(\begin{array}{c} N+r-1\\ r \end{array}\right)=\left(\begin{array}{c} 23\\ 4 \end{array}\right)=8855$$

distinct residue quadruplet types. Each point is generally shared as a common vertex by numerous adjacent tetrahedra in a tessellation; hence the quadruplets defined by these tetrahedra share a common residue. To ensure that each tetrahedron identified an interacting residue quadruplet, tetrahedral edges longer than 12 angstroms were removed from all protein structure tessellations prior to analysis [42].

We previously developed a four-body, knowledge-based statistical contact potential by tessellating a diverse, representative subset of the PDB [27], consisting of 1375 non-redundant (< 30% sequence identity), high-resolution (≤ 2.2 angstroms) x-ray crystallographic protein structures culled using the PISCES server [43]. For each of the 8855 distinct residue quadruplet types (*i*,*j*,*k*,*l*), an observed relative frequency of occurrence *f_ijkl _*was calculated as the proportion of all tetrahedra collectively generated by the tessellations for which the given residue quadruplet appears at the four vertices. A rate expected by chance for the quadruplet was obtained with the use of a multinomial reference distribution, given by

$$p_{ijkl}=\frac{4!}{\overset{20}{\underset{n=1}{\prod }}\left(t_{n}!\right)}\overset{20}{\underset{n=1}{\prod }}a_{n}^{t_{n}}\text{,where }\overset{20}{\underset{n=1}{\sum }}a_{n}=1\text{ and }\overset{20}{\underset{n=1}{\sum }}t_{n}=4.$$

In the above formula, *a_n _*represents the proportion of all amino acids in the tessellated proteins that are of type *n*, and *t_n _*represents the number of occurrences of amino acid *n *in the quadruplet. Through an application of the inverted Boltzmann principle, the log-likelihood score *s_ijkl _= *- log (*f_ijkl _/ p_ijkl_*) was used to quantify an energy of interaction for the residue quadruplet [44]. The collection of 8855 distinct types of residue quadruplets and their respective scores defines the four-body statistical potential.

Returning to the native HIV-1 PR and RT protein structures, each constituent tetrahedron in their tessellations was assigned a score equivalent to that of the residue quadruplet identified at its four vertices. For each tessellation, the sum of all tetrahedral scores defined a *total potential *for the respective protein [27]. A *residue environment score *was also calculated for each PR and RT sequence position by identifying all tetrahedra that share the corresponding point as a vertex and adding up their scores, with the respective 99D (for PR) and 543D (for RT) vectors of residue environment scores referred to as protein *potential profiles *[27,45]. Similar results were obtained for each PR or RT mutational pattern, first by removing the residue identities from the vertices of the respective protein tessellation while retaining their sequence position numbers, then by threading the mutant protein sequence onto those vertices and recalculating the tetrahedral scores. We refer to the difference between mutant and wild-type total potentials as the mutant *residual score*, which quantifies the relative change to protein sequence-structure compatibility; their component-wise potential profile difference as the mutant *residual profile*; and the individual components of a residual profile as *environmental change (EC) scores*, which collectively quantify all sequence position-specific perturbations relative to wild-type [27].

### Prediction algorithms and performance evaluation

Entire residual profiles, as well as distinct subsets of their full complement of EC score components, were employed as the attributes of mutant feature vectors for structure-based models. To each inhibitor dataset, we applied two classification [random forest (RF) [46] and support vector machine (SVM) [47]] and two regression [reduced-error pruned tree (REPTree) regression [48] and support vector regression (SVR) [49]] statistical learning methods for classifying the corresponding mutants into one of three categories. All four algorithms were implemented using the Weka [48] suite of machine learning tools using default parameters, with the following exceptions: for RF, we used forests consisting of 100 trees; for REPTree, we initially performed ten iterations of bootstrap aggregating (bagging) [50] on each dataset; for SVM, we fit logistic models to the outputs in order to obtain proper probability estimates; and for both SVM and SVR, we used a radial basis function (RBF) kernel, we performed neither normalization nor standardization of the mutant attributes, and we varied both the complexity parameter and the RBF gamma parameter in order to optimize performance. For our sequence-based models, each inhibitor-specific dataset of mutants was represented in two distinct ways based on n-grams, according to the type of input attributes used for generating mutant feature vectors (relative frequency versus counts approaches), and we specifically focused on applying RF classification and REPTree regression algorithms with parameters identical to those described above.

Stratified tenfold cross-validation (10-fold CV) testing was used to evaluate the performance of each algorithm on each dataset. For classification models, prediction results were reported by calculating the overall accuracy (ACC, proportion correct), the balanced error rate (BER), the area (AUC) under the receiver operating characteristic (ROC) curve, and the RF out-of-bag (OOB) error rate. For regression models, we reported the Pearson's correlation coefficient between actual and predicted drug susceptibility values for the dataset mutants, the mean-squared error (mse), and the overall accuracy of mutant classifications based on their predicted values. Statistical significance results (*p*-values) were calculated based on the use of appropriate *t*-tests. Additional details regarding these evaluation metrics are available in [28].

## List of abbreviations used

HAART: highly active antiretroviral therapy; PR: protease; RT: reverse transcriptase; PI: protease inhibitor; NRTI: nucleoside/nucleotide RT inhibitor; NNRTI: nonnucleoside RT inhibitor; RF: random forest; SVM: support vector machine; REPTree: reduced-error pruned tree regression; SVR: support vector regression; PDB: Protein Data Bank; EC: environmental change/perturbation; TPV: tipranavir; ddC: zalcitabine; FTC: emtricitabine; IAS: International Antiviral Society; TSM: nonpolymorphic treatment selected mutation; BER: balanced error rate; AUC: area under the receiver operating characteristic curve; ROC: receiver operating characteristic curve; OOB: out-of-bag error; mse: mean-squared error; RTV: ritonavir; d4T: stavudine; NVP: nevirapine; ACC: overall accuracy; 3D: three-dimensional; RBF: radial basis function; 10-fold CV: tenfold cross-validation.

## Competing interests

The authors declare that they have no competing interests.

## Authors' contributions

This study was conceived and managed by IIV. MM performed the experiments and data analysis, generated the tables and figures, and drafted the manuscript. IIV and MM read and approved the final manuscript.

## Supplementary Material

Additional file 1 BER and AUC performance measures associated with RF and SVM classification, using TSM, All, and IAS sets to construct mutant feature vectors Click here for file

Additional file 2 RF classification OOB values, using TSM, All, and IAS sets to construct mutant feature vectors Click here for file

Additional file 3 *r*^2 ^and mse performance measures associated with REPTree and SVR regression, using TSM, All, and IAS sets to construct mutant feature vectors Click here for file

## Declarations

Publication of this article was funded in part by the George Mason University Libraries Open Access Publishing Fund.

This article has been published as part of *BMC Genomics *Volume 14 Supplement S4, 2013: Selected articles from the IEEE International Conference on Bioinformatics and Biomedicine 2012: Genomics. The full contents of the supplement are available online at http://www.biomedcentral.com/bmcgenomics/supplements/14/S4.

## References

[B1] Broder S The development of antiretroviral therapy and its impact on the HIV-1/AIDS pandemic Antiviral Res 2010 14 1 18 10.1016/j.antiviral.2009.10.002 20018391 PMC2815149

[B2] Panel on Antiretroviral Guidelines for Adults and Adolescents. Department of Health and Human Services: Guidelines for the use of antiretroviral agents in HIV-1-infected adults and adolescents http://www.aidsinfo.nih.gov/ContentFiles/AdultandAdolescentGL.pdf

[B3] Ren J Nichols C Bird L Chamberlain P Weaver K Short S Stuart DI Stammers DK Structural mechanisms of drug resistance for mutations at codons 181 and 188 in HIV-1 reverse transcriptase and the improved resilience of second generation non-nucleoside inhibitors J Mol Biol 2001 14 795 805 10.1006/jmbi.2001.4988 11575933

[B4] Johnson VA Brun-Vezinet F Clotet B Gunthard HF Kuritzkes DR Pillay D Schapiro JM Richman DD Update of the drug resistance mutations in HIV-1: December 2010 Top HIV Med 2010 14 156 163 21245516

[B5] Shafer RW Schapiro JM HIV-1 drug resistance mutations: an updated framework for the second decade of HAART AIDS Rev 2008 14 67 84 18615118 PMC2547476

[B6] Schmidt B Walter H Moschik B Paatz C van Vaerenbergh K Vandamme AM Schmitt M Harrer T Uberla K Korn K Simple algorithm derived from a geno-/phenotypic database to predict HIV-1 protease inhibitor resistance AIDS 2000 14 1731 1738 10.1097/00002030-200008180-00007 10985309

[B7] Zazzi M Romano L Venturi G Shafer RW Reid C Dal Bello F Parolin C Palu G Valensin PE Comparative evaluation of three computerized algorithms for prediction of antiretroviral susceptibility from HIV type 1 genotype J Antimicrob Chemother 2004 14 356 360 10.1093/jac/dkh021 14688053

[B8] Wang K Jenwitheesuk E Samudrala R Mittler JE Simple linear model provides highly accurate genotypic predictions of HIV-1 drug resistance Antivir Ther 2004 14 343 352 15259897

[B9] Puchhammer-Stockl E Steininger C Geringer E Heinz FX Comparison of virtual phenotype and HIV-SEQ program (Stanford) interpretation for predicting drug resistance of HIV strains HIV Med 2002 14 200 206 10.1046/j.1468-1293.2002.00116.x 12139659

[B10] DiRienzo AG DeGruttola V Larder B Hertogs K Non-parametric methods to predict HIV drug susceptibility phenotype from genotype Stat Med 2003 14 2785 2798 10.1002/sim.1516 12939786

[B11] Beerenwinkel N Schmidt B Walter H Kaiser R Lengauer T Hoffmann D Korn K Selbig J Diversity and complexity of HIV-1 drug resistance: a bioinformatics approach to predicting phenotype from genotype Proc Natl Acad Sci USA 2002 14 8271 8276 10.1073/pnas.112177799 12060770 PMC123057

[B12] Beerenwinkel N Daumer M Oette M Korn K Hoffmann D Kaiser R Lengauer T Selbig J Walter H Geno2pheno: estimating phenotypic drug resistance from HIV-1 genotypes Nucleic Acids Res 2003 14 3850 3855 10.1093/nar/gkg575 12824435 PMC168981

[B13] Wang D Larder B Enhanced prediction of lopinavir resistance from genotype by use of artificial neural networks J Infect Dis 2003 14 653 660 10.1086/377453 12934180

[B14] Chen YZ Gu XL Cao ZW Can an optimization/scoring procedure in ligand-protein docking be employed to probe drug-resistant mutations in proteins? J Mol Graph Model 2001 14 560 570 10.1016/S1093-3263(01)00091-2 11552685

[B15] Shenderovich MD Kagan RM Heseltine PN Ramnarayan K Structure-based phenotyping predicts HIV-1 protease inhibitor resistance Protein Sci 2003 14 1706 1718 10.1110/ps.0301103 12876320 PMC2323957

[B16] Stoffler D Sanner MF Morris GM Olson AJ Goodsell DS Evolutionary analysis of HIV-1 protease inhibitors: Methods for design of inhibitors that evade resistance Proteins 2002 14 63 74 10.1002/prot.10130 12012338

[B17] Chen X Weber IT Harrison RW Molecular dynamics simulations of 14 HIV protease mutants in complexes with indinavir J Mol Model 2004 14 373 381 10.1007/s00894-004-0205-x 15597206

[B18] Draghici S Potter RB Predicting HIV drug resistance with neural networks Bioinformatics 2003 14 98 107 10.1093/bioinformatics/19.1.98 12499299

[B19] Ravich VL Masso M Vaisman II A combined sequence-structure approach for predicting resistance to the non-nucleoside HIV-1 reverse transcriptase inhibitor Nevirapine Biophys Chem 2011 14 168 172 10.1016/j.bpc.2010.11.004 21146283

[B20] Kjaer J Hoj L Fox Z Lundgren JD Prediction of phenotypic susceptibility to antiretroviral drugs using physiochemical properties of the primary enzymatic structure combined with artificial neural networks HIV Med 2008 14 642 652 10.1111/j.1468-1293.2008.00612.x 18631257

[B21] Masso M Vaisman II Yang J, Yang M, Zhu M, Zhang Y, Arabnia H, Deng Y, Bourbakis N A novel sequence-structure approach for accurate prediction of resistance to HIV-1 protease inhibitors Proceedings of the 7th IEEE International Conference on Bioinformatics and Bioengineering 2007 Boston 952 958 IEEE

[B22] Prosperi MC Rosen-Zvi M Altmann A Zazzi M Di Giambenedetto S Kaiser R Schulter E Struck D Sloot P van de Vijver DA Vandamme AM Sonnerborg A Antiretroviral therapy optimisation without genotype resistance testing: a perspective on treatment history based models PLoS One 2010 14 e13753 10.1371/journal.pone.0013753 21060792 PMC2966424

[B23] Zazzi M Incardona F Rosen-Zvi M Prosperi M Lengauer T Altmann A Sonnerborg A Lavee T Schulter E Kaiser R Predicting response to antiretroviral treatment by machine learning: the EuResist project Intervirology 2012 14 123 127 10.1159/000332008 22286881

[B24] Rhee SY Taylor J Wadhera G Ben-Hur A Brutlag DL Shafer RW Genotypic predictors of human immunodeficiency virus type 1 drug resistance Proc Natl Acad Sci USA 2006 14 17355 17360 10.1073/pnas.0607274103 17065321 PMC1622926

[B25] Stanford University HIV Drug Resistance Database http://hivdb.stanford.edu/

[B26] Berman HM Westbrook J Feng Z Gilliland G Bhat TN Weissig H Shindyalov IN Bourne PE The protein data bank Nucleic Acids Res 2000 14 235 242 10.1093/nar/28.1.235 10592235 PMC102472

[B27] Masso M Vaisman II Accurate prediction of enzyme mutant activity based on a multibody statistical potential Bioinformatics 2007 14 3155 3161 10.1093/bioinformatics/btm509 17977887

[B28] Masso M Prediction of human immunodeficiency virus type 1 drug resistance: representation of target sequence mutational patterns via an n-grams approach Bioinformatics and Biomedicine (BIBM), 2012 IEEE International Conference on: 4-7 October 2012 2012 1 6 10.1109/BIBM.2012.6392665

[B29] Lin LI A concordance correlation coefficient to evaluate reproducibility Biometrics 1989 14 255 268 10.2307/2532051 2720055

[B30] Johnson VA Brun-Vezinet F Clotet B Conway B Kuritzkes DR Pillay D Schapiro J Telenti A Richman D Update of the drug resistance mutations in HIV-1: 2005 Top HIV Med 2005 14 51 57 15849371

[B31] Rhee SY Fessel WJ Zolopa AR Hurley L Liu T Taylor J Nguyen DP Slome S Klein D Horberg M Flamm J Follansbee S Schapiro JM Shafer RW HIV-1 protease and reverse-transcriptase mutations: correlations with antiretroviral therapy in subtype B isolates and implications for drug-resistance surveillance J Infect Dis 2005 14 456 465 10.1086/431601 15995959 PMC2597526

[B32] Dong Q Zhou S Deng L Guan J Gene ontology-based protein function prediction by using sequence composition information Protein Pept Lett 2010 14 789 795 10.2174/092986610791190336 19995340

[B33] Vries JK Liu X Bahar I The relationship between n-gram patterns and protein secondary structure Proteins 2007 14 830 838 10.1002/prot.21480 17523186

[B34] Cheng BY Carbonell JG Klein-Seetharaman J Protein classification based on text document classification techniques Proteins 2005 14 955 970 10.1002/prot.20373 15645499

[B35] Mansoori EG Zolghadri MJ Katebi SD Protein superfamily classification using fuzzy rule-based classifier IEEE Trans Nanobioscience 2009 14 92 99 19307166 10.1109/TNB.2009.2016484

[B36] Zhang KX Ouellette BF GAIA: a gram-based interaction analysis tool--an approach for identifying interacting domains in yeast BMC Bioinformatics 2009 14 S60 19208164 10.1186/1471-2105-10-S1-S60 PMC2648738

[B37] Petropoulos CJ Parkin NT Limoli KL Lie YS Wrin T Huang W Tian H Smith D Winslow GA Capon DJ Whitcomb JM A novel phenotypic drug susceptibility assay for human immunodeficiency virus type 1 Antimicrob Agents Chemother 2000 14 920 928 10.1128/AAC.44.4.920-928.2000 10722492 PMC89793

[B38] Zhang J Rhee SY Taylor J Shafer RW Comparison of the precision and sensitivity of the Antivirogram and PhenoSense HIV drug susceptibility assays J Acquir Immune Defic Syndr 2005 14 439 444 10.1097/01.qai.0000147526.64863.53 15764961 PMC2547471

[B39] Parkin NT Hellmann NS Whitcomb JM Kiss L Chappey C Petropoulos CJ Natural variation of drug susceptibility in wild-type human immunodeficiency virus type 1 Antimicrob Agents Chemother 2004 14 437 443 10.1128/AAC.48.2.437-443.2004 14742192 PMC321508

[B40] Barber CB Dobkin DP Huhdanpaa HT The quickhull algorithm for convex hulls ACM Trans Math Software 1996 14 469 483 10.1145/235815.235821

[B41] de Berg M Cheong O van Kreveld M Overmars M Computational Geometry: Algorithms and Applications 2008 Berlin: Springer-Verlag

[B42] Masso M Vaisman II Accurate prediction of stability changes in protein mutants by combining machine learning with structure based computational mutagenesis Bioinformatics 2008 14 2002 2009 10.1093/bioinformatics/btn353 18632749

[B43] Wang G Dunbrack RL Jr PISCES: a protein sequence culling server Bioinformatics 2003 14 1589 1591 10.1093/bioinformatics/btg224 12912846

[B44] Sippl MJ Boltzmann's principle, knowledge-based mean fields and protein folding. An approach to the computational determination of protein structures Journal of Computer-Aided Molecular Design 1993 14 473 501 10.1007/BF02337562 8229096

[B45] Bowie JU Luthy R Eisenberg D A method to identify protein sequences that fold into a known three-dimensional structure Science 1991 14 164 170 10.1126/science.1853201 1853201

[B46] Breiman L Random forests Machine Learning 2001 14 5 32 10.1023/A:1010933404324

[B47] Platt J Schoelkopf B, Burges C, Smola A Fast training of support vector machines using sequential minimal optimization Advances in Kernel Methods-Support Vector Learning 1998 Cambridge: MIT Press 185 208

[B48] Frank E Hall M Trigg L Holmes G Witten IH Data mining in bioinformatics using Weka Bioinformatics 2004 14 2479 2481 10.1093/bioinformatics/bth261 15073010

[B49] Smola AJ Scholkopf B A tutorial on support vector regression http://www.svms.org/regression/SmSc98.pdf

[B50] Breiman L Bagging predictors Machine Learning 1996 14 123 140

