# Supplementary material for: Sequence and structure based models of HIV-1 protease and reverse transcriptase drug resistance
Source: BMC Genomics. 2013 Oct 1;14(Suppl 4):S3. doi: 10.1186/1471-2164-14-S4-S3 (PMC3849442; doi:10.1186/1471-2164-14-S4-S3)
Supplement: Additional file 1 — BER and AUC performance measures associated with RF and SVM classification, using TSM, All, and IAS sets to construct mutant feature vectors [file 1471-2164-14-S4-S3-S1.pdf]

Additional file 1. BER and AUC performance measures associated with RF and SVM classification, using TSM, All, and IAS sets to construct mutant feature vectors

Drug	RF						SVM						Drug Mean	
	TSM		All		IAS		TSM		All		IAS			
	BER	AUC	BER	AUC	BER	AUC	BER	AUC	BER	AUC	BER	AUC	BER	AUC
Protease Inhibitors														
APV	0.32	0.91	0.32	0.92	0.31	0.91	0.38	0.88	0.35	0.89	0.37	0.86	0.34	0.90
ATV	0.29	0.90	0.29	0.89	0.29	0.88	0.40	0.82	0.41	0.79	0.42	0.80	0.35	0.85
IDV	0.28	0.93	0.28	0.93	0.27	0.93	0.29	0.90	0.29	0.91	0.28	0.88	0.28	0.91
LPV	0.25	0.94	0.24	0.94	0.26	0.94	0.34	0.88	0.35	0.89	0.34	0.88	0.30	0.91
NFV	0.22	0.93	0.20	0.94	0.22	0.92	0.24	0.90	0.23	0.90	0.24	0.90	0.23	0.92
RTV	0.18	0.96	0.18	0.96	0.19	0.96	0.26	0.92	0.29	0.92	0.25	0.92	0.23	0.94
SQV	0.27	0.94	0.26	0.95	0.26	0.94	0.27	0.93	0.26	0.93	0.27	0.92	0.27	0.94
TPV	0.61	0.95	0.67	0.94	0.61	0.90	0.31	0.80	0.37	0.86	0.21	0.91	0.46	0.89
AVG	0.30	0.93	0.31	0.93	0.30	0.92	0.31	0.88	0.32	0.89	0.30	0.88	0.31	0.91
Nucleoside / Nucleotide RT Inhibitors														
3TC	0.16	0.99	0.18	0.98	0.17	0.98	0.21	0.96	0.18	0.95	0.21	0.96	0.19	0.97
ABC	0.29	0.86	0.29	0.85	0.29	0.85	0.31	0.83	0.34	0.80	0.33	0.82	0.31	0.84
AZT	0.31	0.91	0.31	0.89	0.30	0.90	0.33	0.88	0.26	0.91	0.34	0.87	0.31	0.89
d4T	0.23	0.90	0.29	0.89	0.31	0.87	0.23	0.89	0.23	0.85	0.28	0.89	0.26	0.88
ddC	0.24	0.88	0.29	0.88	0.22	0.89	0.32	0.83	0.30	0.84	0.32	0.82	0.28	0.86
ddI	0.29	0.86	0.33	0.86	0.30	0.80	0.25	0.85	0.30	0.79	0.26	0.80	0.29	0.83
FTC	0.14	1.00	0.14	1.00	0.00	1.00	0.19	0.91	0.25	0.88	0.14	0.91	0.14	0.95
TDF	0.45	0.87	0.46	0.89	0.50	0.77	0.47	0.78	0.44	0.76	0.56	0.61	0.48	0.78
AVG	0.26	0.91	0.29	0.91	0.26	0.88	0.29	0.87	0.29	0.85	0.31	0.84	0.28	0.87
Nonnucleoside RT Inhibitors														
DLV	0.32	0.87	0.35	0.88	0.35	0.86	0.32	0.86	0.35	0.84	0.32	0.87	0.34	0.86
EFV	0.20	0.94	0.25	0.92	0.21	0.92	0.28	0.88	0.33	0.85	0.32	0.87	0.27	0.90
NVP	0.31	0.91	0.35	0.92	0.29	0.90	0.36	0.86	0.41	0.82	0.35	0.87	0.35	0.88
AVG	0.28	0.91	0.32	0.91	0.28	0.89	0.32	0.87	0.36	0.84	0.33	0.87	0.32	0.88
