# Supplementary material for: Sequence and structure based models of HIV-1 protease and reverse transcriptase drug resistance
Source: BMC Genomics. 2013 Oct 1;14(Suppl 4):S3. doi: 10.1186/1471-2164-14-S4-S3 (PMC3849442; doi:10.1186/1471-2164-14-S4-S3)
Supplement: Additional file 2 — RF classification OOB values, using TSM, All, and IAS sets to construct mutant feature vectors [file 1471-2164-14-S4-S3-S2.pdf]

Additional file 2. RF classification OOB values, using TSM, All, and IAS sets to construct mutant feature vectors

	RF			
Drug	TSM	All	IAS	Drug Mean
		Protease Inhibitors		
APV	0.24	0.20	0.23	0.22
ATV	0.29	0.26	0.32	0.29
IDV	0.23	0.23	0.24	0.23
LPV	0.23	0.21	0.23	0.22
NFV	0.21	0.19	0.23	0.21
RTV	0.14	0.16	0.16	0.15
SQV	0.18	0.17	0.19	0.18
TPV	0.21	0.19	0.21	0.20
AVG	0.22	0.20	0.23	0.21
		Nucleoside / Nucleotide RT Inhibitors		
3TC	0.13	0.15	0.11	0.13
ABC	0.33	0.34	0.30	0.32
AZT	0.26	0.25	0.26	0.26
d4T	0.23	0.24	0.28	0.25
ddC	0.20	0.24	0.22	0.22
ddI	0.26	0.24	0.27	0.26
FTC	0.08	0.10	0.08	0.09
TDF	0.29	0.26	0.33	0.29
AVG	0.22	0.23	0.23	0.23
		Nonnucleoside RT Inhibitors		
DLV	0.24	0.26	0.27	0.26
EFV	0.18	0.21	0.18	0.19
NVP	0.17	0.16	0.14	0.16
AVG	0.20	0.21	0.20	0.20
