# Supplementary material for: Sequence and structure based models of HIV-1 protease and reverse transcriptase drug resistance
Source: BMC Genomics. 2013 Oct 1;14(Suppl 4):S3. doi: 10.1186/1471-2164-14-S4-S3 (PMC3849442; doi:10.1186/1471-2164-14-S4-S3)
Supplement: Additional file 3 — r2 and mse performance measures associated with REPTree and SVR regression, using TSM, All, and IAS sets to construct mutant feature vectors [file 1471-2164-14-S4-S3-S3.pdf]

Additional file 3. r^2 and mse performance measures associated with REPTree and SVR regression, using TSM, All, and IAS sets to construct mutant feature vectors

Drug	REPTree						SVR						Drug Mean	
	TSM		All		IAS		TSM		All		IAS			
	r^2	mse	r^2	mse	r^2	mse	r^2	mse	r^2	mse	r^2	mse	r^2	mse
Protease Inhibitors														
APV	0.70	0.24	0.71	0.23	0.70	0.23	0.62	0.29	0.59	0.31	0.58	0.32	0.65	0.27
ATV	0.61	0.30	0.68	0.26	0.62	0.30	0.59	0.31	0.58	0.31	0.51	0.37	0.60	0.31
IDV	0.78	0.17	0.78	0.18	0.76	0.18	0.73	0.20	0.73	0.20	0.70	0.23	0.75	0.19
LPV	0.78	0.24	0.77	0.24	0.79	0.22	0.69	0.34	0.69	0.34	0.64	0.38	0.73	0.29
NFV	0.79	0.21	0.80	0.19	0.77	0.22	0.77	0.23	0.75	0.25	0.73	0.26	0.77	0.23
RTV	0.81	0.22	0.84	0.18	0.78	0.25	0.77	0.26	0.80	0.23	0.68	0.37	0.78	0.25
SQV	0.69	0.37	0.70	0.35	0.66	0.40	0.75	0.29	0.77	0.26	0.71	0.34	0.71	0.34
TPV	0.44	0.36	0.50	0.35	0.45	0.34	0.48	0.30	0.52	0.28	0.64	0.22	0.51	0.31
AVG	0.70	0.26	0.72	0.25	0.69	0.27	0.68	0.28	0.68	0.27	0.65	0.31	0.69	0.27
Nucleoside / Nucleotide RT Inhibitors														
3TC	0.96	0.04	0.95	0.05	0.96	0.04	0.90	0.18	0.90	0.12	0.90	0.18	0.93	0.10
ABC	0.77	0.04	0.75	0.04	0.78	0.04	0.73	0.04	0.74	0.04	0.65	0.06	0.74	0.04
AZT	0.67	0.38	0.70	0.36	0.61	0.45	0.74	0.29	0.72	0.32	0.65	0.41	0.68	0.37
d4T	0.70	0.03	0.70	0.03	0.70	0.03	0.71	0.03	0.70	0.03	0.75	0.02	0.71	0.03
ddC	0.64	0.04	0.62	0.04	0.63	0.04	0.67	0.04	0.60	0.05	0.65	0.04	0.64	0.04
ddI	0.73	0.02	0.73	0.02	0.67	0.02	0.70	0.02	0.74	0.02	0.65	0.02	0.70	0.02
FTC	0.98	0.03	0.97	0.04	0.98	0.03	0.76	0.31	0.64	0.50	0.89	0.15	0.87	0.18
TDF	0.48	0.05	0.50	0.05	0.26	0.07	0.50	0.05	0.55	0.04	0.18	0.08	0.41	0.06
AVG	0.74	0.08	0.74	0.08	0.70	0.09	0.71	0.12	0.70	0.14	0.67	0.12	0.71	0.10
Nonnucleoside RT Inhibitors														
DLV	0.64	0.49	0.63	0.49	0.64	0.49	0.65	0.49	0.57	0.58	0.68	0.44	0.64	0.50
EFV	0.72	0.37	0.75	0.35	0.72	0.38	0.68	0.44	0.58	0.58	0.67	0.46	0.69	0.43
NVP	0.76	0.40	0.78	0.37	0.77	0.38	0.76	0.41	0.59	0.69	0.73	0.46	0.73	0.45
AVG	0.71	0.42	0.72	0.40	0.71	0.42	0.70	0.45	0.58	0.62	0.69	0.45	0.69	0.46
